# Surgical and Conservative Management of Periorbital Tumors in Four Dogs

**DOI:** 10.3390/vetsci13070714

**Published:** 2026-07-20

**Authors:** Iosif Vasiu, Mădălina Florina Dragomir, Ciprian Andrei Ober, Cosmin Petru Peştean, Romelia Pop, Marian Taulescu, Claudiu Gal, Seppo Saari, David Vicente

**Affiliations:** 1 Department of Anesthesiology and Surgery, Faculty of Veterinary Medicine, University of Agricultural Sciences and Veterinary Medicine of Cluj-Napoca, 3-5 Mănăştur Street, 400372 Cluj-Napoca, Romania; madalina.dragomir@usamvcluj.ro (M.F.D.); ciprian.ober@usamvcluj.ro (C.A.O.); cosmin.pestean@usamvcluj.ro (C.P.P.); 2 Department of Pathology, Faculty of Veterinary Medicine, University of Agricultural Sciences and Veterinary Medicine of Cluj-Napoca, 3-5 Mănăştur Street, 400372 Cluj-Napoca, Romania; romelia.pop@usamvcluj.ro (R.P.); marian.taulescu@usamvcluj.ro (M.T.); 3 Synevovet, 81 Pache Protopopescu, 021408 Bucharest, Romania; gal.claudiu@gmail.com; 4 Eläinpatologian Laboratorio Patovet, 00151 Helsinki, Finland; seppo@patovet.fi; 5 Department of Surgery, Veterinary Hospital Mevet, 4 Hiomotie Street, 00380 Helsinki, Finland; david.g.vicente@gmail.com; 6 Egas Moniz School of Health & Science, Campus Universitário, Quinta da Granja Street, 2829-511 Caparica, Portugal; 7 Veterinary Clinic VetAmadora, 3 Salvador Allende Street, 2700-042 Amadora, Portugal

**Keywords:** excision, dog, periorbital, neoplasia, zygomatic, sebaceous gland carcinoma, multilobular tumor of bone, inflammatory myofibroblastic tumor

## Abstract

Four client-owned dogs with histopathologically confirmed periorbital tumors were included in this retrospective case series: one spayed female, one intact male, and two neutered males. The dogs ranged in age from 6 to 8 years. The most common clinical signs were slowly progressive exophthalmos (4/4 dogs), epiphora (3/4 dogs), and prolapse of the nictitating membrane gland (2/4 dogs). Periorbital disease was initially suspected based on the clinical examination. Computed tomography (CT) was subsequently used to characterize the presence, location, and extent of the lesion and for surgical planning, whereas histopathological examination confirmed the tumor type. The identified tumors included two multilobular tumors of bone (MTB), one inflammatory myofibroblastic tumor (IMT), and one sebaceous carcinoma (SC). Treatment for the first three dogs consisted of wide tumor excision via combined modified lateral orbitotomy and partial ostectomies of the zygomatic arch and mandibular ramus. The remaining dog received conservative therapy. For the first two surgically treated dogs, survival times were 55 months; the third dog was lost to follow-up two weeks after surgery. For the conservatively treated dog, the survival time was 4 months. Although limited by the small number of heterogeneous cases, this report suggests that the described surgical approach facilitated *en bloc* excision in these three surgically treated dogs. However, histologically tumor-free surgical margins were confirmed in only one of the three surgically treated dogs, and clinical outcomes varied according to tumor type, extent of disease, and duration of follow-up. Therefore, further studies are required before definitive conclusions regarding oncologic outcome or prognosis can be drawn.

## Simple Summary

Treatment of canine periorbital tumors is challenging due to their heterogeneity, making surgical margins crucial. In this case series, we examine three types of periorbital masses—multilobular tumors of bone (MTB), inflammatory myofibroblastic tumor (IMT), and sebaceous carcinoma (SC)—after treatment. Wide excision with combined lateral orbitotomy and partial osteotomies of the zygomatic arch and mandibular ramus was performed on two dogs with MTBs and one with IMT, while a dog with SC received chemotherapy. Surgery enabled *en bloc* excision of the lesions in all three surgically treated dogs; however, complete histologic excision (histologically tumor-free surgical margins) was achieved in only one case, whereas incomplete surgical margins were identified in the remaining two dogs. Two dogs had follow-ups over 50 months; the dog on chemotherapy survived only four months.

## 1. Introduction

The orbitozygomaticomaxillary complex (OZMC), formed by the zygomatic, maxillary, frontal, lacrimal and palatine bones, represents one of the most anatomically complex regions of the canine skull. Besides its essential role in mastication and facial conformation, this region contains numerous critical neurovascular structures and forms the bony framework of the orbit, making the diagnosis, imaging evaluation and surgical management of lesions particularly challenging [1].

Periorbital and orbital tumors are uncommon but clinically important because they frequently invade adjacent anatomical structures, including the retrobulbar space, eyelids, nasal cavity, paranasal sinuses and oral cavity. In advanced cases, progressive bone destruction may allow intracranial extension through the cranial vault or adjacent neurovascular foramina [2,3,4,5,6,7,8]. As a consequence, affected dogs commonly present with slowly progressive exophthalmos, epiphora, prolapse of the gland of the third eyelid, conjunctival hyperemia, facial swelling or impaired ocular motility. Since these clinical signs are often initially subtle and may mimic inflammatory orbital disease, diagnosis is frequently delayed until the tumors become locally advanced, reducing the likelihood of achieving complete surgical excision [2,3,4,5,6,7,8].

Canine orbital neoplasia comprises a wide spectrum of histological diagnoses, including epithelial, mesenchymal, neuroectodermal, round-cell and adipocytic tumors [9]. Although biologically benign, lipomas and other adipose tumors may also produce clinically important orbital disease because of their space-occupying effect. Among malignant tumors, MTB, IMT and SC represent uncommon entities with markedly different biological behaviour, prognosis and therapeutic requirements [7,10,11,12,13,14,15,16,17,18,19,20,21,22,23,24,25,26]. Although complete surgical excision remains the treatment of choice whenever feasible, obtaining adequate oncologic margins in the periorbital region is particularly difficult because of the limited surgical exposure and the close relationship with the globe, optic nerve, extraocular muscles and major neurovascular structures [1,4,5,6].

Several orbitotomy techniques have been described to improve access to orbital lesions, including lateral orbitotomy, modified lateral orbitotomy and more extensive orbitectomy procedures [4,5,27,28,29,30]. Recently, combining modified lateral orbitotomy with partial ostectomies of the zygomatic arch and mandibular ramus has been proposed to increase surgical exposure while preserving masticatory function and facilitating *en bloc* resection of selected invasive tumors [31]. Nevertheless, published clinical experience with this approach remains limited, particularly for uncommon periorbital tumor types.

The purpose of the present case series was not to compare tumor biology but to illustrate the clinical presentation, diagnostic work-up, surgical decision-making and outcomes of four dogs with uncommon periorbital tumors managed using different therapeutic strategies. Particular emphasis is placed on the practical application, advantages and limitations of combined modified lateral orbitotomy with partial zygomatic arch and mandibular ramus ostectomies for achieving *en bloc* excision of selected invasive periorbital tumors, while highlighting how preoperative imaging, tumor extent and ocular involvement influenced surgical planning and globe management.

## 2. Description of Cases

Four dogs with histopathologically confirmed periorbital tumors were retrospectively reviewed. For each case, clinical presentation, diagnostic work-up, CT findings, surgical planning, operative management, histopathological diagnosis, postoperative care and long-term outcome were evaluated at the Small Animal Emergency Hospital in Cluj-Napoca (Romania) and one at the Veterinary Hospital Mevet in Helsinki (Finland) (Table 1). Tissue specimens, fixed in neutral buffered formalin, were collected and submitted for histopathology for all four cases. Moreover, a fine needle aspirate (FNA) was collected only from the first dog. A board-certified pathologist reviewed all the specimens.

In all four cases, telephone follow-up was assessed monthly, and the owners were instructed to return to the veterinary hospital for re-evaluation at three-month intervals for the first year after surgery.

### 2.1. Case 1

**Figure 1:**
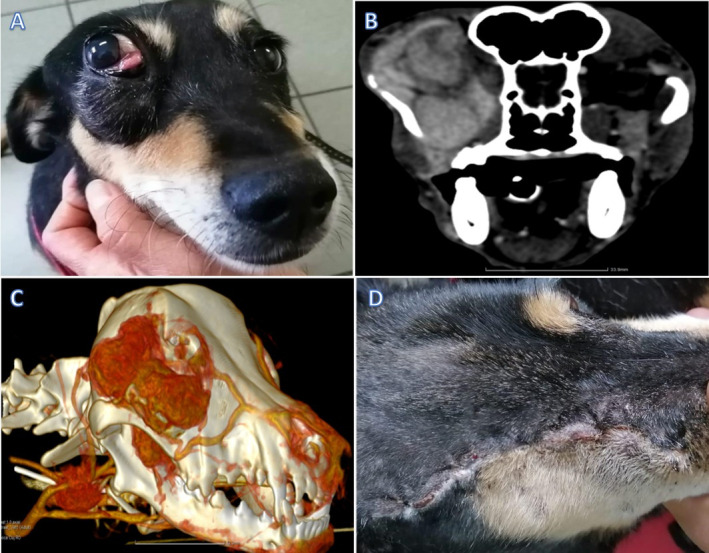
Case 1. (**A**) Clinical aspect of the right periorbital area before the surgical intervention. (**B**) A preoperative axial view of the CT scan highlights the presence of a soft tissue attenuating mass along the right periorbital area invading the eye globe. (**C**) A preoperative three-dimensional rendering of the dog’s skull illustrating the zygomatic bone. (**D**) Clinical aspect of the right periorbital area three weeks after the surgical intervention.

The first case was a 14.4 kg, 8-year-old neutered mixed-breed dog from a dog shelter, referred for a slow-growing zygomatic arch mass and secondary exophthalmos of the right eye (Figure 1A). An initial physical examination at the hospital revealed exophthalmos, conjunctival hyperemia, epiphora, and prolapse of the nictitating membrane gland.

**Figure 2:**
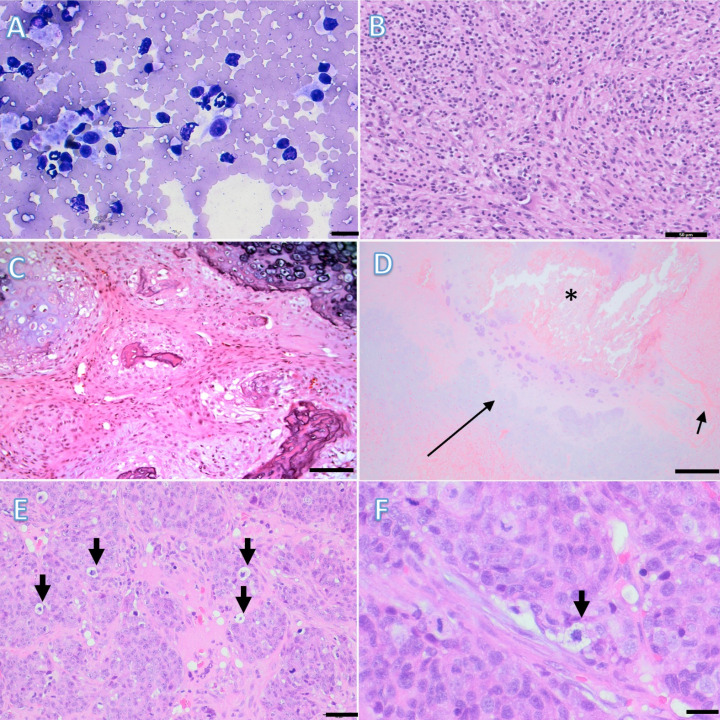
Cytological and histopathological interpretation of the periorbital tumors. Case 1. (**A**). Initial cytological examination revealed a spindled to stellate cell proliferation, with rare, fine intracytoplasmic granular pigment, admixed with rare neutrophils and small lymphocytes. Scale bar = 20 µm. May–Grünwald Giemsa; (**B**). Subsequent histological examination revealed a myofibroblastic proliferation admixed with inflammatory cells, consistent with an inflammatory myofibroblastic tumor. Scale bar = 50 µm. H&E; Case 2. (**C**). A micrograph showing variably sized and shaped islands with central mineralized cartilage separated by septae of mesenchymal cells. Scale bar = 100 µm. H&E; Case 3. (**D**). Typical elements of a multilobular tumor of bone: Variable-sized islands of cartilage (long arrow) with central mineralization (*) and osteoid formation (small arrows). Scale bar = 200 µm. H&E; Case 4. (**E**). Incisional biopsies revealed an epithelial malignant proliferation, composed of nests and lobules of epithelial cells, with occasional sebaceous differentiation (arrows), consistent with a sebaceous carcinoma. Scale bar = 50 µm. H&E; (**F**). Higher magnification of sebaceous differentiation of the neoplastic cells (arrow). Scale bar = 20 µm. H&E.

Additionally, the complete blood count (CBC, VetScan, Abaxis, York, UK), biochemistry (Element RC, Scil, Viernheim, Germany), and abdominal ultrasound (US; MyLabDeltaVet, Esaote, Fishers, IN, USA) results were unremarkable. Contrast-enhanced CT (SOMATOM Scope, Siemens, Erlangen, Germany) demonstrated invasion of the globe, retrobulbar tissues, temporal bone and zygomatic arch (Figure 1B,C). Because of direct ocular involvement and the extent of the lesion, globe preservation was not considered feasible and exenteration was planned as part of the oncologic resection. Thoracic radiographs and abdominal US revealed no evidence of metastases. The dog handler elected for an FNA from the retrobulbar mass before the surgery. The cytological evaluation indicated the presence of a putative amelanotic melanoma (Figure 2A).

Two months after the first consultation, a wide tumor excision via combined modified lateral orbitotomy and partial ostectomies of the zygomatic arch and mandibular ramus was performed. Ceftriaxone was administered (Cefort 1 g, Antibiotice Iaşi, Romania; 22 mg/kg IV) preoperatively and then every 60 min throughout the procedure. Wide surgical margins (approximately 2 cm) were planned based on CT findings. Following skin incision, the temporalis and masseter muscles were elevated to expose the zygomatic arch. Partial ostectomies of the zygomatic arch and dorsal mandibular ramus were performed using an oscillating saw to improve surgical access. Exenteration was carried out because CT demonstrated direct invasion of the globe and retrobulbar tissues. The tumor was dissected *en bloc* together with the affected orbital contents while preserving major surrounding neurovascular structures whenever possible. Surgical margins of approximately 2 cm were planned whenever anatomically feasible, although histologically tumor-free surgical margins could not be achieved because of invasion of adjacent skull structures. Exenteration was required because CT demonstrated direct invasion of the globe and retrobulbar tissues, precluding globe preservation. The surgical field was copiously lavaged before routine layered closure using advancement of local soft tissues. The recovery from surgery was uneventful. No intraoperative complications, including excessive hemorrhage or injury to adjacent neurovascular structures, were encountered. Surgical planning based on CT imaging allowed safe exposure of the retrobulbar compartment and *en bloc* tumor excision despite extensive local invasion. The dog was discharged home approximately 24 h following surgery with robenacoxib (Onsior 2%, Elanco GmbH, Germany; 2 mg/kg SC SID for 5 days) and clavulanate amoxicillin (Synulox 250 mg, Zoetis, Belgium; 15 mg/kg SC SID for 5 days). Postoperative pain was managed using a multimodal analgesic protocol based on opioids during hospitalization followed by non-steroidal anti-inflammatory drugs after discharge. Analgesia was adjusted according to repeated clinical assessment of patient comfort, appetite, activity, and response to handling during hospitalization. Although a formal validated pain-scoring system was not used retrospectively, no patient required rescue analgesia prior to discharge.

After surgery, the entire excised specimen was submitted for histopathological evaluation. Histopathological examination did not confirm the initial cytological suspicion of an amelanotic melanoma but instead established the diagnosis of an IMT. Gross examination of the excised specimen revealed a mass measuring 5 × 2 cm (gross measurement). Histopathological evaluation demonstrated incomplete histologic excision (histologically incomplete surgical margins) (Figure 2B).

The dog handler was instructed to feed only a canned and soft kibble diet, to avoid toys or mouth play, restrict the dog’s exercise to short-lead walks and place an Elizabethan collar for 14 days.

**Figure 3:**
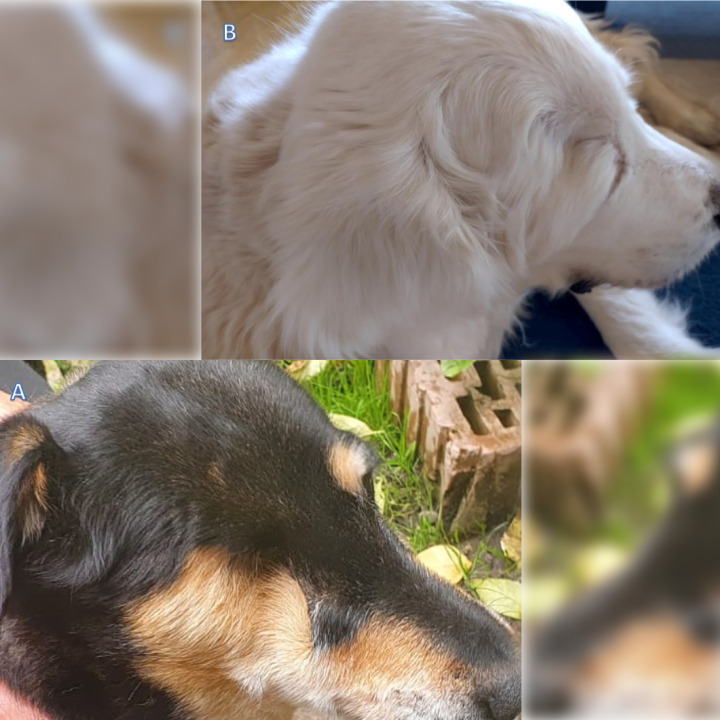
Postsurgical outcome. (**A**) Case 1: postoperative appearance at follow-up. (**B**) Case 2: postoperative appearance at follow-up. (**A**) Clinical aspect of the right periorbital area of the first dog, at 12 months after surgery. (**B**) Clinical aspect of the right periorbital area of the second dog, at 55 months after surgery.

The skin sutures were removed 14 days after discharge. The surgical site healed with a good cosmetic outcome (Figure 1D). The dog handler reported normal prehension and behavior. The dog was bright and alert, tolerated food and water, and walked three times daily. The dog was reevaluated 55 months postoperatively. Clinical examination revealed no gross evidence of local tumor recurrence (Figure 3A), and the owner reported no changes in eating, drinking, or behavior. However, no follow-up CT examination or repeat oncologic staging was performed; therefore, residual disease or subclinical local recurrence could not be definitively excluded, particularly considering the histologically incomplete surgical margins. The dog subsequently died following a clinical diagnosis of renal insufficiency. No necropsy was performed; therefore, metastatic disease or tumor-related causes of death could not be completely excluded.

### 2.2. Case 2

**Figure 4:**
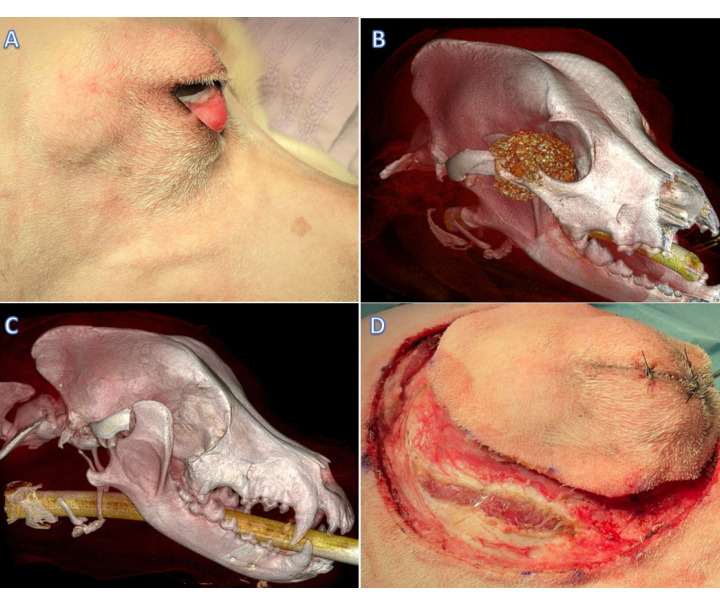
Case 2. (**A**) Clinical aspect of the right periorbital area before the surgical intervention. (**B**) Preoperative three-dimensional rendering of the dog’s skull portraying the zygomatic mass. (**C**) Postoperative three-dimensional rendering of the dog’s skull portrays the extent of the zygomatic excision. (**D**) Intraoperative view of wide excision of an intraorbital mass with exenteration of the eye and partial zygomatic arch excision.

The second case was a 42.3 kg, 8-year-old castrated Golden Retriever male, referred for a mass in the right caudal zygomatic arch region, causing secondary exophthalmos and prolapse of the nictitating membrane of the eye (Figure 4A). One month previously, the primary care veterinarian evaluated the dog for right-sided prolapse of the nictitating membrane gland. It was surgically repositioned using Morgan’s pocket technique, but it relapsed five days following surgery. Thus, the patient was referred to a veterinary ophthalmologist, who identified a hard mass over the zygomatic arch and exophthalmos of the right eye. An initial physical examination at the hospital revealed moderate exophthalmos, conjunctival hyperemia, and prolapse of the nictitating membrane gland of the right eye.

Although menace response and pupillary light reflexes indicated retained vision, CT demonstrated marked retrobulbar extension of the mass. Globe-sparing surgery was considered unlikely to permit adequate oncologic margins or safe *en bloc* excision; therefore, orbital exenteration was elected. Additionally, the CBC (Idexx ProCyte Dx Hematology Analyzer, IDEXX Laboratories, Westbrook, ME, USA), biochemistry (Catalyst Dx Chemistry Analyzer, IDEXX Laboratories, Westbrook, ME, USA), and abdominal US (Logiq F6, GE Healthcare, Wauwatosa and Waukesha, WI, USA) results were unremarkable. Contrast-enhanced CT (SOMATOM Scope, Siemens, Erlangen, Germany) of the head confirmed the presence of a multilobular mass with a bone density arising from the right zygomatic bone extending rostrally into the orbital space, causing exophthalmos (Figure 4B,C). Thoracic radiographs and abdominal US revealed no evidence of metastases. The owner elected for an excisional biopsy for economic reasons, and no other staging information was obtained.

**Figure 5:**
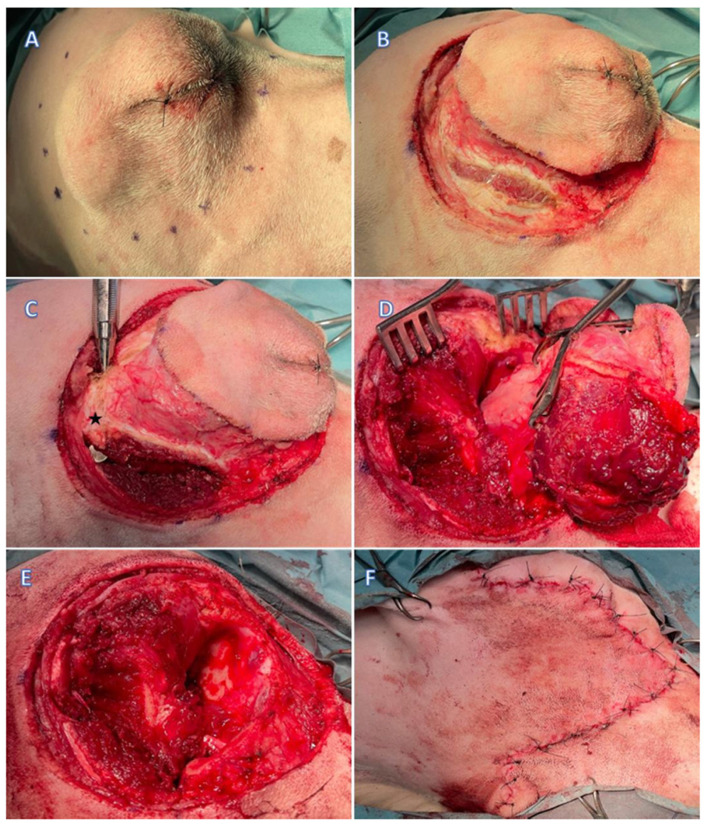
Surgical management of Case 2 (multilobular tumor of bone). (**A**). Preoperative marking of wide surgical margins (approximately 2 cm) around the right periorbital mass; (**B**). Elliptical incision through the skin and subcutaneous tissue in the periorbital region; (**C**). Isolation of the zygomatic bone (star) to allow for osteotomy; (**D**). Periorbital dissection and exenteration of the right eye and associated orbital contents; (**E**). Block excision of the right eye and periorbital region; (**F**). Primary closure of the defect with a single pedicle advancement flap.

A wide tumor excision, including modified lateral orbitotomy and partial ostectomies of the zygomatic arch and mandibular ramus, was performed (Figure 5A–F and Figure 4D). Wide surgical margins (approximately 2 cm) were planned according to the CT findings. Following elevation of the temporalis and masseter muscles, partial ostectomies of the zygomatic arch and dorsal mandibular ramus were performed to improve surgical exposure. The orbital mass was removed *en bloc* together with the orbital contents while preserving adjacent neurovascular structures whenever possible. Routine layered closure was subsequently performed after copious lavage of the surgical field. Intraoperative visualization of the retrobulbar compartment was considered excellent after temporary removal of the zygomatic arch and partial mandibular ostectomy, facilitating controlled dissection around the tumor while minimizing unnecessary manipulation of adjacent tissues.

Cefazolin (Zolinef 1 g, Medochemie Ltd., Cyprus; 22 mg/kg IV TID) was administered intraoperatively and then every 60 min throughout the procedure. The dog remained hospitalized for approximately 24 h, during which multimodal analgesia, supportive care, and serial clinical evaluations were performed before discharge with carprofen (Rimadyl 50 mg, Zoetis, Italy; PO BID for 10 days), paracetamol (Paramax 250 mg, Vitabalans Pharma OÜ, Finland; 6 mg/kg PO BID for 5 days) and cephalexin (Kefavet 500 mg, Orion Pharma, Finland; 11 mg/kg PO BID for 5 days). After surgery, the entire mass was sent for histopathological evaluation. Histopathological examination confirmed a grade I MTB measuring 4 × 3 cm (gross measurement), with histologically tumor-free surgical margins. The minimum histologic margin measured less than 1 mm (Figure 2C).

The owner was instructed to feed only a canned and soft kibble diet, to avoid toys or mouth play, restrict the dog’s exercise to short-lead walks and place an Elizabethan collar for 14 days.

The skin sutures were removed 14 days after discharge. The surgical site healed with a good cosmetic outcome. No postoperative wound complications, dehiscence, infection, or impairment of food prehension or mastication were reported. The owner reported normal prehension and behavior. The dog was bright and alert, tolerated food and water, and walked three times daily. The dog was reevaluated 55 months postoperatively. Clinical examination revealed no gross evidence of tumor recurrence (Figure 3B), and the owner reported no changes in eating, drinking, or behavior. However, no follow-up CT examination or repeat cross-sectional imaging was performed; therefore, residual disease or subclinical local recurrence could not be definitively excluded despite the favorable long-term clinical outcome.

### 2.3. Case 3

**Figure 6:**
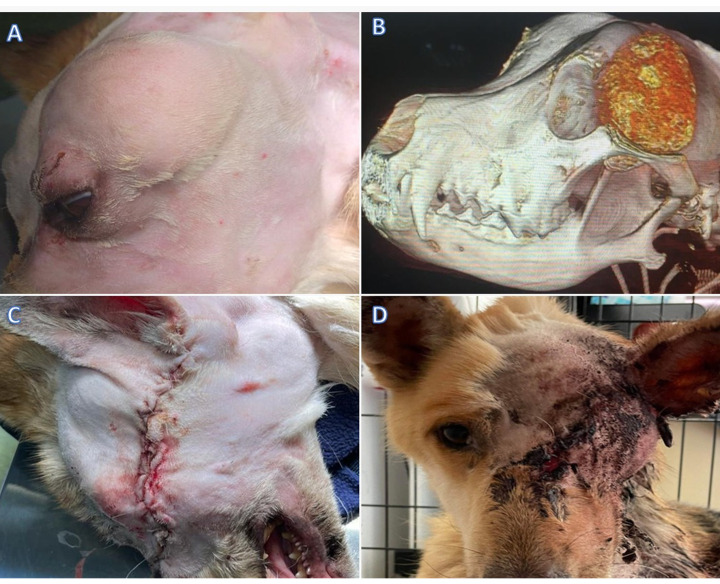
Case 3. (**A**) Clinical aspect of the left periorbital area before the surgical intervention. (**B**) Preoperative three-dimensional rendering of the dog’s skull portraying the zygomatic mass. (**C**) Clinical aspect of the left periorbital area after the surgical intervention. (**D**) Clinical aspect of the suture site at 14 days after surgery.

The third case was an 18 kg, 6-year-old spayed female mixed-breed dog from a dog shelter, referred for a large caudal zygomatic arch mass and secondary exophthalmos of the left eye (Figure 6A). An initial physical examination at the hospital revealed exophthalmos and epiphora. Additionally, the CBC revealed lymphocytosis, alongside monocytosis and neutrophilia, with normochromic-normocytic anemia.

Furthermore, decreased levels of Ca (6,5; 11 ± 0.52 mg/dL), albumin (1.3; 2.5–3.7 g/dL), total protein (TP; 5.4; 5.5–8.5 g/dL), glycemia (60.69; 62–117 mg/dL), and increased globulin levels (4.2; 2.1–4.0 g/dL) were detected. A Contrast-enhanced CT of the head confirmed the presence of an invasive ovoid and multilobular orbital mass affecting the superior maxilla, orbit, and left zygomatic arch (Figure 6B). Given the extensive retrobulbar involvement and the need to maximize surgical margins, globe preservation was not considered compatible with the planned oncologic resection, and exenteration was performed.

Thoracic radiographs and abdominal US revealed no evidence of metastases. The dog handler elected to undergo an excisional biopsy for economic reasons, and no other staging information was obtained.

One month after the first consultation, a wide tumor excision via combined modified lateral orbitotomy and partial ostectomies of the zygomatic arch and mandibular ramus was performed (Figure 6C). Wide surgical margins (approximately 2 cm) were planned preoperatively based on CT findings whenever anatomically feasible. After elevation of the temporalis and masseter muscles, partial ostectomies of the zygomatic arch and dorsal mandibular ramus were performed to improve exposure of the retrobulbar compartment. The mass and orbital contents were excised *en bloc*. Routine layered closure was performed after copious lavage of the surgical field. Despite extensive local invasion, the combined surgical approach provided sufficient exposure to permit *en bloc* excision while preserving the surrounding major neurovascular structures whenever anatomically possible. Ceftriaxone was administered (22 mg/kg IV) preoperatively and then every 60 min throughout the procedure. The recovery from surgery was uneventful, and the dog recovered quickly and satisfactorily. Postoperative pain was managed using a multimodal analgesic protocol based on opioids during hospitalization followed by non-steroidal anti-inflammatory drugs after discharge. The dog was discharged home approximately 24 h postop with robenacoxib (2 mg/kg SC SID for 5 days) and clavulanate amoxicillin (15 mg/kg SC SID for 5 days). After surgery, the entire mass was sent for histopathological evaluation. An MTB mass 5 x 8 cm (gross measurement) without clean surgical margins was confirmed (Figure 2D).

The dog handler was instructed to feed only a canned and soft kibble, diet to avoid toys or mouth play, restrict the dog’s exercise to short-lead walks and place an Elizabethan collar for 14 days.

The skin sutures were removed 14 days after discharge. The surgical site healed with a good cosmetic outcome (Figure 6D). No postoperative wound complications, dehiscence, infection, or impairment of food prehension or mastication were observed during the available follow-up period. The dog handler reported normal prehension and behavior. The dog was bright and alert, tolerated food and water, and walked three times daily. The dog was lost to further follow-up.

### 2.4. Case 4

**Figure 7:**
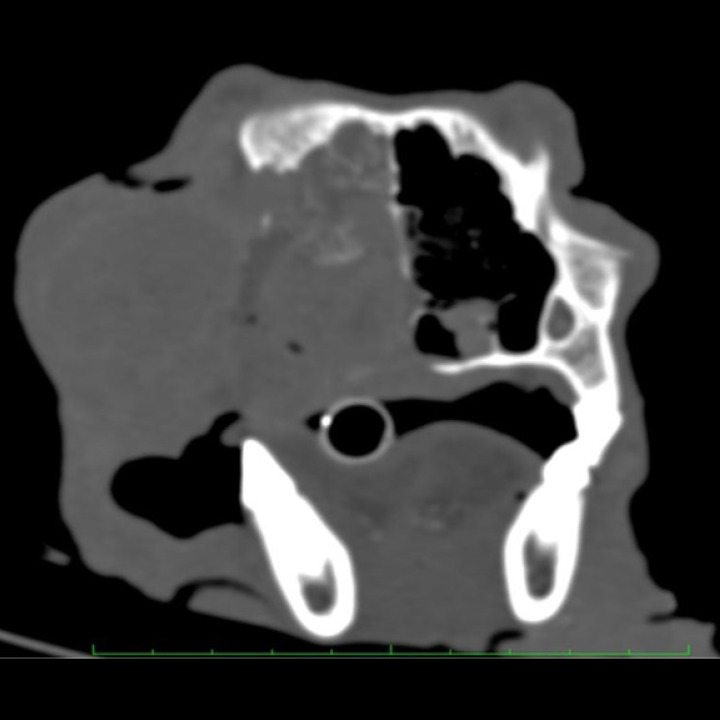
Case 4. Axial view of the CT scan depicting a soft tissue attenuating mass along the left periorbital area.

The fourth case was a 30 kg, 8-year-old intact Transylvanian Hound male, referred for a rapidly growing and invasive, 5 cm diameter left zygomatic mass (Figure 7). The primary care veterinarian initially evaluated the dog for an approximately two-week history of exophthalmos and mucopurulent epiphora of the left eye and prescribed antibiotic (clavulanate amoxicillin; 20 mg/kg SC BID) and anti-inflammatory (carprofen; 3 mg/kg SC SID) therapy. The mass initially reduced in size but grew again following cessation of the antibiotic course. Additionally, radiographs performed at the local veterinarian showed the presence of periorbital bone lysis, and though it could not be excluded, without any evidence of pulmonary metastases.

An initial physical examination at the hospital showed moderate exophthalmos and an elastic cutaneous mass with mucopurulent epiphora. Additionally, the CBC indicated lymphopenia (0.62; 1–4.8 × 10^9^/L), hyperproteinemia (7.7; 5–7.2 mg/dL), and decreased urea (18.0; 19.7–62.5 mg/dL) levels. Contrast-enhanced CTof the head showed a soft tissue mass that had aggressively invaded the left nasal cavity, causing complete obstruction, and severe osteolysis affecting the left nasal bone and the bony palate (Figure 7). The soft tissue structure extended into the surrounding structures, invading the left periorbital area. The CT scan also identified several small mineralized structures within the mass (Figure 7).

A biopsy of the mass was performed, and the sample obtained was sent for histopathology evaluation. The histopathology of the tissue sample confirmed the presence of periorbital high-malignant SC with lymphatic invasion (Figure 2E,F).

The owner chose chemotherapy with carboplatin, 300 mg/m2 IV q4wk. Antibiotic therapy (cephalexin; 22 mg/kg SC SID, metronidazole15 mg/kg IV BID) and anti-inflammatory (piroxicam; 0.3 mg/kg SID) management was done by the referring veterinarian. Radical surgical excision was not recommended because CT demonstrated extensive invasion of the nasal cavity, palate and surrounding facial structures, making complete oncologic resection unlikely. The dog was euthanized four months after the initial presentation because of progressive local disease, deterioration in quality of life (QOL), and the owner’s decision to avoid further suffering. No repeat CT examination, oncologic staging, or necropsy was performed before or after euthanasia. Consequently, the extent of local progression, possible intracranial extension, or distant metastatic disease could not be determined. Therefore, the reported survival time should be interpreted cautiously and cannot be used to draw conclusions regarding prognosis or treatment efficacy.

Overall, the objective of the case descriptions was not to compare biological behavior among tumor types but to illustrate how preoperative imaging, ocular involvement, and local tumor extension influenced therapeutic decision-making and surgical planning in individual patients.

## 3. Discussion

In this retrospective case series, we describe the clinical presentation, diagnostic work-up, surgical management and clinical outcome of four dogs with uncommon periorbital tumors, including IMT, MTB and SC. Rather than comparing the biological behaviour of these distinct tumor types, the principal objective of this report was to illustrate how preoperative imaging, local tumor extension and ocular involvement influenced therapeutic decision-making and the application of a combined modified lateral orbitotomy with partial zygomatic arch and mandibular ramus ostectomies for *en bloc* excision of selected invasive lesions. Diagnostic evaluation consisted of clinical examination, contrast-enhanced CT of the head, thoracic imaging for metastatic screening, histopathological confirmation in all dogs, and abdominal ultrasonography in the surgically managed cases. These findings emphasize the value of individualized treatment planning based on tumor extent rather than histologic diagnosis alone [1,3,4,5,6,9,32].

All four dogs presented with slowly progressive exophthalmos, whereas epiphora and prolapse of the gland of the third eyelid were also common findings. These observations agree with previous reports describing orbital tumors as slowly expanding space-occupying lesions that progressively displace the globe and surrounding periocular structures [3,4,5,6,8]. Although these clinical signs are not pathognomonic, they should prompt further imaging because inflammatory orbital disease and neoplasia frequently present with similar ophthalmic abnormalities during the early stages of disease [3,5,6].

Contrast-enhanced CT proved essential for defining the extent of local invasion, identifying osseous involvement, evaluating globe invasion and planning the surgical approach in all four dogs. In each surgically managed case, CT findings directly influenced the decision to perform orbital exenteration because preservation of the globe would have compromised oncologic resection. Although globe-sparing surgery may be appropriate in carefully selected dogs with localized orbital or periorbital tumors, preservation of a visual and comfortable eye should not compromise adequate oncologic excision. Therefore, the decision between globe preservation and exenteration must be individualized according to tumor extent, direct globe invasion, visual status, ocular comfort, and the likelihood of obtaining acceptable surgical margins. In the present series, exenteration was considered necessary because CT demonstrated either direct globe invasion or extensive retrobulbar involvement that precluded conservative orbital surgery. Histopathology ultimately established the definitive diagnosis in every patient, whereas cytology was performed only in Case 1 and yielded a presumptive diagnosis that differed from the final histopathological diagnosis. This finding supports previous reports indicating that cytology alone may be insufficient for definitive characterization of orbital tumors and reinforces the importance of histopathological examination for accurate diagnosis [3,5,9,16,33,34].

The principal clinical contribution of this case series is the practical application of a combined modified lateral orbitotomy with partial ostectomies of the zygomatic arch and mandibular ramus for the management of extensive periorbital tumors. In all three surgically treated dogs, this approach substantially improved visualization of the retrobulbar compartment and permitted controlled *en bloc* excision despite extensive local invasion. Nevertheless, adequate surgical exposure should not be interpreted as synonymous with complete oncologic excision. Histologically tumor-free margins were achieved in only one dog, whereas the remaining two cases demonstrated histologically incomplete excision because tumor infiltration extended beyond anatomically resectable boundaries. These findings illustrate that even when exposure is optimized, complete margin control remains constrained by tumor biology and regional anatomy [4,5,6,27,28,29,30,31,32].

Clinical outcomes reflected the heterogeneous biological behaviour of the different tumor types rather than the surgical technique itself. Both dogs available for long-term clinical follow-up remained free of gross clinical recurrence for approximately 55 months. However, neither underwent follow-up CT nor repeat oncologic staging; consequently, residual disease or subclinical local recurrence could not be excluded, particularly in the dog with histologically incomplete surgical margins. Similarly, metastatic disease could not be definitively excluded in Case 1 because necropsy was not performed following death from clinically diagnosed renal insufficiency. The third dog was lost to follow-up shortly after surgery, whereas the fourth dog underwent conservative treatment because extensive local invasion precluded radical resection and was euthanized because of progressive disease. Accordingly, survival data should be interpreted cautiously and not as evidence of treatment efficacy or prognosis.

Several limitations should be acknowledged. The study includes only four dogs with different tumor types, preventing meaningful comparison of biological behaviour or therapeutic outcome. Surgical margins differed among cases, postoperative imaging was unavailable during long-term follow-up, and complete oncologic staging or necropsy was not performed in all patients. Consequently, local recurrence and metastatic disease cannot be definitively excluded despite favorable clinical outcomes in some dogs. These limitations are inherent to retrospective case series involving uncommon tumors and should be considered when interpreting the results.

## 4. Conclusions

This case series illustrates the diagnostic and therapeutic management of four dogs with uncommon periorbital tumors presenting with different biological behaviors and clinical outcomes. Contrast-enhanced CT proved essential for defining tumor extent, assessing ocular and osseous involvement, guiding surgical planning, and supporting treatment selection. In carefully selected cases, combined modified lateral orbitotomy with partial ostectomies of the zygomatic arch and mandibular ramus provided excellent surgical exposure and facilitated *en bloc* excision of extensive periorbital tumors. However, optimized surgical exposure did not consistently result in histologically tumor-free surgical margins, emphasizing the importance of individualized surgical planning, thorough ophthalmic evaluation, margin assessment, and long-term oncologic follow-up. Because this retrospective series included only four heterogeneous cases and was limited by incomplete postoperative imaging, variable follow-up, and the absence of necropsy in some patients, no definitive conclusions regarding recurrence, survival, or treatment efficacy can be drawn. Larger prospective studies are warranted to further evaluate the long-term oncologic outcomes of this surgical approach.

## Figures and Tables

**Table 1:** History, diagnosis, follow-up, outcome and survival time.

	Breed	Age (Years)	Sex	Weight (kg)	Reproductive Status	Tumor Topography	Imaging	Diagnostic	Follow-Up (Days)	Outcome
1	Mix-breed *	8	M	14.4	Castrated male	Right periorbital area (OD)	CT	IMT″	55 mo (1697)	No recurrence
2	Golden Retriever ^	8	M	42.3	Castrated male	Right periorbital area (OD)	CT	MTB′	55 mo (1681)	No recurrence
3	Mix-breed *	6	F	18.0	Spayed female	Left periorbital area (OS)	CT	MTB″	2 weeks (14)	Lost to follow-up
4	Transylvanian Hound *	7.2	M	30.0	Intact male	Left periorbital area (OS)	CT	SC	4 mo (122)	Euthanized

M—male; F—female; CT—computed tomography; IMT—Inflammatory myofibroblastic tumor; MTB—Multilobular tumor of bone; SC—Sebaceous carcinoma; OD—*oculus dexter*; OS *oculus sinister*; mo—months. * Dogs diagnosed and treated in Romania. ^^^ Dog diagnosed and treated in Finland. ′ Histologically tumor-free surgical margins were confirmed by the histopathology report. ″ Histologically incomplete surgical margins were identified on histopathological examination.

## Data Availability

The datasets used and/or analyzed during the current study are available from the corresponding author upon reasonable request.

## Abbreviations

The following abbreviations are used in this manuscript:

BID	Twice daily
Ca	Calcium
CBC	Complete blood count
CT	Computed tomography
F	Female
FNA	Fine needle aspirate
H&E	Hematoxylin and Eosin
IMT	Inflammatory myofibroblastic tumor
IV	Intravenously
M	Male
Mo	Months
MTB	Multilobular tumor of bone
*od*	*oculus dexter*
*os*	*oculus sinister*
OZMC	Orbitozygomaticomaxillary complex
PO	Per os
q4wk	Every 4 weeks
QOL	Quality of life
SC	Sebaceous carcinoma
SC	Subcutaneous/Subcutaneously
SID	Once daily
TP	Total proteins
US	Ultrasound
WBC	White blood cell
